# Laser-Induced Graphene on Novel Crosslinked Poly(dimethylsiloxane)/Triton X-100 Composites for Improving Mechanical, Electrical and Hydrophobic Properties

**DOI:** 10.3390/polym16223157

**Published:** 2024-11-13

**Authors:** Marija V. Pergal, Milena Rašljić Rafajilović, Teodora Vićentić, Igor A. Pašti, Sanja Ostojić, Danica Bajuk-Bogdanović, Marko Spasenović

**Affiliations:** 1 Center for Microelectronic Technologies, Institute of Chemistry, Technology and Metallurgy, National Institute of the Republic of Serbia, University of Belgrade, Njegoševa 12, 11000 Belgrade, Serbia; milena.rasljic@ihtm.bg.ac.rs (M.R.R.); teodora.vicentic@ihtm.bg.ac.rs (T.V.); marko.spasenovic@ihtm.bg.ac.rs (M.S.); 2 Faculty of Physical Chemistry, University of Belgrade, Studentski trg 12-16, 11158 Belgrade, Serbiadanabb@ffh.bg.ac.rs (D.B.-B.); 3 The Institute of General and Physical Chemistry, Studentski trg 12/V, 11158 Beograd, Serbia

**Keywords:** poly(dimethylsiloxane), polymer composites, laser-induced graphene, CO_2_ laser, material characterization

## Abstract

Laser-induced graphene (LIG) has become a highly promising material for flexible functional devices due to its robust mechanical stability, excellent electrical properties, and ease of fabrication. Most research has been focused on LIG production on rigid or flexible substrates, with an obvious gap in laser induction of graphene on elastic, stretchable substrates, which limits the scope of application of LIG in flexible electronics. We demonstrate laser induction of graphene on a novel, cross-linked poly(dimethylsiloxane) (PDMS)/Triton X-100 composite substrates. The effect of varying Triton content (1–30 wt.%) on the structural, thermal, surface, nanomechanical, and electrical properties of LIG was systematically studied. Physicochemical characterization confirmed the successful induction of LIG on the surface of PDMS/Triton composites. A higher content of Triton in the PDMS matrix improves the quality of LIG, increases stiffness and hydrophobicity, and somewhat decreases sheet resistance. Similar thermal properties and super-hydrophobicity were observed for LIG/PDMS/Triton materials as compared to their counterparts without LIG. Direct laser irradiation of graphene on the surface of PDMS/Triton composites results in the formation of extremely promising materials, which have great potential for use in flexible electronic devices.

## 1. Introduction

Flexible electronics is an emerging field of research and industry that integrates conductive materials with polymers or metal substrates [1,2]. Traditional conductors lack flexibility, whereas conventional elastomers, such as polyurethane (PU), poly(dimethylsiloxane) (PDMS), natural rubber (NR), and butadiene rubber (SBR), are not conductive [1,2]. As research and development of flexible electronics progresses, the demand for suitable materials for device fabrication is increasing. Several studies reported materials exhibiting excellent combined elasticity and conductivity, but these benefits often come at the cost of complex fabrication processes, intricate patterning strategies, or non-environmentally friendly synthesis methods for active materials [3,4,5,6,7].

Recently, laser-induced graphene (LIG), owing to its superior electrical and mechanical properties, cost-effectiveness, ease of fabrication, piezoresistivity, and high surface-to-volume ratio, has emerged as a highly promising material for fabricating various flexible electronic devices [8,9,10,11,12]. LIG consists of a three-dimensional network of few-layer graphene produced with the laser-writing process on a polymeric surface [9]. This process primarily involves carbonization and graphenization of the polymer film surface by using a laser [9,13]. By adjusting various processing parameters, LIG with distinct morphological characteristics can be fabricated [13]. This process’s versatility and adaptability have resulted in applications across multiple disciplines, including electronics, catalysis, and energy storage [14,15,16,17,18,19,20,21]. The graphenization mechanism of polymers is strongly correlated with the physicochemical characteristics and structural features of the repeat units, particularly aromatic and imide repeat units [9].

To prepare LIG as a sensing layer for flexible strain sensors, researchers have predominantly utilized direct laser irradiation on a limited number of polymers, such as polyimide (PI), poly(etherimide), or poly(ether-ether-ketone) (PEEK) [16,22,23,24,25]. Currently, commercial PI film is the most common substrate for producing high-quality, carbon-rich LIG with good thermal and mechanical stability [13]. However, PI has disadvantages, such as suboptimal biocompatibility and stretchability, prompting researchers to explore the transfer of LIG onto various other elastic materials to form composites [19,26,27]. For example, LIG has been transferred to PDMS to produce a flexible device on biocompatible material, directly applied to the skin for monitoring heartbeat [19]. However, the microstructure of LIG can often be damaged during the transfer process, resulting in increased initial resistance or incomplete transfer of the graphene layer, which translates to material loss and potential device damage [10,19].

Despite extensive efforts to develop LIG on new substrates, a dearth of stretchable polymers suitable for laser graphenization remains. In today’s flexible microsystem technology, poly(dimethylsiloxane) (PDMS) is the most popular elastomeric material, due to its attractive physical and chemical properties, including elasticity, optical transparency, tunable surface chemistry, low water permeability, biocompatibility, UV resistance, good thermal and oxidative stability, high gas permeability, and high dielectric properties [28,29]. Furthermore, PDMS is low-cost, non-toxic, and hydrophobic, making it suitable for simple fabrication processes [29]. Its excellent mechanical and chemical properties make PDMS the material of choice for protective layers, microdevices, supercapacitors, and sensors [30,31,32].

One-step fabrication of LIG on flexible precursors appears to be a promising strategy. Parmeggiani et al. [33] developed PI/PDMS elastic composite substrates by mixing PI powder with PDMS, followed by CO_2_ laser irradiation to produce LIG for flexible strain sensors. The particles undergo graphenization during laser writing, but in these composites, conductivity remained limited because of the weal connections between LIG on individual particles, differing from the uninterrupted LIG film achievable on a continuous PI surface [33]. The same authors developed a composite film based on PDMS and triethylene glycol (TEG), which can be directly laser-irradiated to generate 3D porous graphene [34]. Adding TEG as a carbon source to the PDMS matrix improves graphenization and reduces the sheet resistance of the LIG on PDMS/TEG composites. Tang et al. [8] prepared a composite of PEEK powder and PDMS to fabricate flexible electrodes using direct laser-induced graphenization, with promising applications in wearable devices. Over time, the range of precursors for LIG has expanded to include pure polymers with structures similar to PI [35,36,37,38], as well as other materials like sodium alginate, lignin, wood, paper, and certain foods [39,40,41,42]. Generally, manufacturing parameters can be categorized into material composition (e.g., the weight ratio of PI, PEEK, or TEG to PDMS) [8,33,34] and laser scanning conditions (e.g., laser scan speed, laser power, frequency, and resolution) [39]. These parameters significantly influence the electrical and mechanical performance of LIG-based polymeric substrates.

This study presents the first-ever formation of LIG on crosslinked PDMS/Triton X-100 composite films. Triton X-100 was added to the PDMS matrix as a carbon and aromatic ring source to enhance the graphenization of PDMS. Triton X-100 enhances polymer film conductivity by stabilizing nanosized polymer particles and regulating the hydrophile-lipophile balance and critical micelle concentration due to its amphiphilic properties [2,43,44]. It also acts as a plasticizer and inhibits the PDMS crosslinking reaction [44]. A series of PDMS/Triton composite films with different contents of Triton X-100 (1–30 wt.%) in the PDMS matrix was prepared to identify the optimal composition for graphene production on their surfaces through laser induction. Direct laser induction of graphene on the surface of crosslinked PDMS/Triton composite materials opens new possibilities for inscribing electrical circuits, such as for sensors and flexible electronic devices. The LIG produced was intensively studied in order to provide broad and detailed information on its physicochemical characteristics. Various characterization methods, i.e., Fourier transform infrared (FTIR) spectroscopy, Raman spectroscopy, X-ray diffraction (XRD) analysis, scanning electron microscopy with energy-dispersive X-ray spectroscopy (SEM-EDX), thermogravimetric analysis (TGA) in both oxygen and nitrogen atmospheres, differential scanning calorimetry (DSC), nanoindentation, water contact angle and sheet resistance measurements, were employed to analyze the structural, surface, thermal, nanomechanical, and electrical properties of the novel LIG materials.

## 2. Experimantal Section

### 2.1. Materials

Poly(dimethylsiloxane) (PDMS; Sylgard 184) was received from Dow Corning (Midland, MI, USA) in combination with a curing agent. Triton X-100 (Ph. Eur. grade) was purchased from Merck (Darmstadt, Germany). All the chemicals were used as received without further purification.

### 2.2. Preparation of Pure PDMS Film

**Figure 1:**
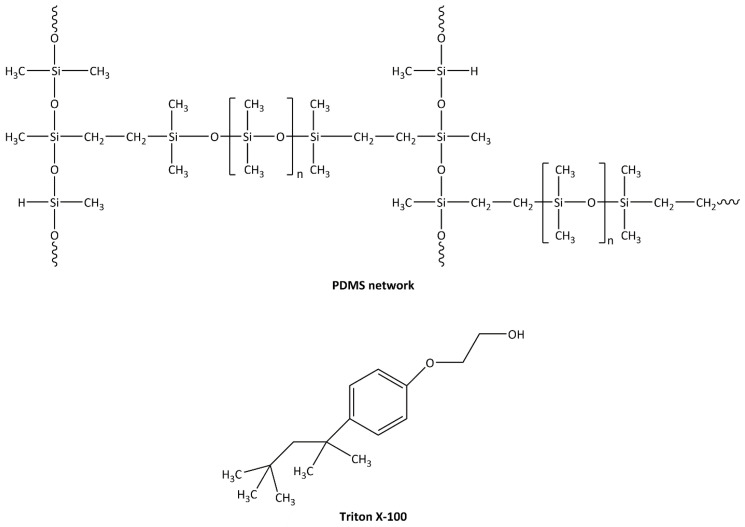
Simplified chemical structures of crosslinked poly(dimethylsiloxane) (PDMS) and Triton X-100.

A pure PDMS network was cast as a film in a mold. The network constituents were α,ω-divinyl-terminated-PDMS (the main component of Part A of Sylgard 184) and poly(methylhydrogensiloxane) (the main component of Part B of Sylgard 184). This siloxane elastomer was prepared via a conventional method, i.e., by a hydrosilylation reaction, maintaining a hydrogen– and vinyl–siloxane ratio of 1:10 (wt:wt). Initially, all components were mixed into a beaker at room temperature on a magnetic stirrer at 100 rpm for 30 min. Subsequently, the mixture was degassed under vacuum for 30 min to remove air bubbles. This mixture was then poured into a mold and crosslinked at 60 °C for 1 h in an oven, followed by a 24 h curing phase in a vacuum oven at 50 °C. After this crosslinking, the final polymer network was obtained, with the presumed structure illustrated in Figure 1. The prepared transparent PDMS film, approximately 2 mm thick, was peeled from the mold and subjected to further analyses.

### 2.3. Preparation of PDMS/Triton Composite Films

A series of PDMS/Triton composite films with different contents of Triton X-100 (1–30 wt.%) was prepared. The PDMS prepolymer and curing agent were mixed in a 10:1 weight ratio, and Triton X-100 was immediately added to the PDMS mixture at predefined concentrations, namely, 1, 5, 10, 20, and 30 wt.%. Each mixture was stirred at room temperature for 30 min to ensure homogeneity and was subsequently degassed for 30 min. Then, the mixtures were poured into molds and crosslinked at 60 °C for 1 h in an oven, followed by curing at 50 °C for 24 h in a vacuum oven. After curing, the PDMS/Triton composite film was gently peeled from the mold. Simplified chemical structures of crosslinked PDMS and Triton X-100 are shown in Figure 1. Films with more than 30 wt.% Triton were very soft and mechanically unstable while being removed from the mold. For that reason, we did not prepare PDMS/Triton composites with more than 30 wt.% Triton in our study.

### 2.4. Laser Writing

**Figure 2:**
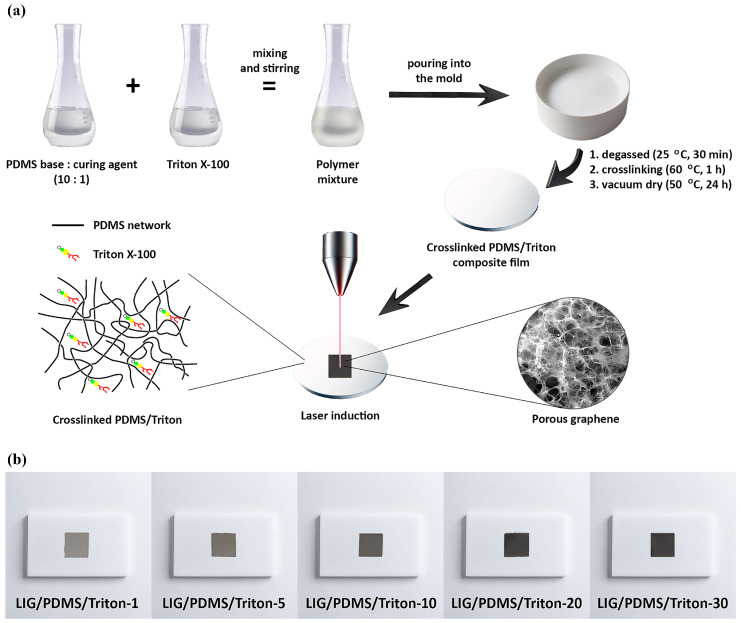
(**a**) Schematic illustration of PDMS/Triton preparation and formation of laser-induced graphene (LIG) on PDMS/Triton composite films. (**b**) Photographs of LIG/PDMS/Triton materials.

LIG was prepared with a CO_2_ laser that has an emission wavelength of 10.6 μm (DBK FL-350; Radlje ob Dravi, Slovenia). The spot size of the laser beam in focus is ~150 μm, and the focal distance is 5 cm. The irradiation was performed in the air. To obtain homogeneous LIG on the PDMS/Triton composite film surfaces, the laser parameters (power, scanning speed, and resolution) were optimized. The laser power was set to 14% (8.4 W of the total available 60 W), which was equivalent to an irradiance of 47 kW cm^−2^. The scanning speed was maintained at a constant rate of 45 mm s^−1^, and the resolution was set at 423 DPI. Figure 2a illustrates the full fabrication process.

### 2.5. Methods of Characterization

Fourier-transform infrared (FTIR) spectra were obtained using attenuated total reflection (ATR) mode on a Nicolet 6700 spectrometer (Thermo Scientific, Waltham, MA, USA) equipped with a diamond crystal and spectra were corrected by the ATR correction. The scanning range was from 4000 to 500 cm^−1^, with a resolution of 4 cm^−1^, and 64 scans were collected for each material.

Raman spectroscopy was conducted using a DXR Raman microscope (Thermo Scientific, USA) equipped with a diode-pumped solid-state laser at an excitation wavelength of 532 nm. The samples were analyzed with a laser power of 2.0 mW, an exposure time of 10 s, 10 exposures per spectrum, a grating with 900 lines mm^−1^, and a 50 μm pinhole spectrograph aperture. The laser beam was focused on the sample using an objective with 10× magnification. From Raman spectra, we calculated crystallite size (L_a_) along the a-axis of graphitic materials according to Equation (1) [45]:

(1) $$L_{a}(nm)=(2.4\times 10^{-10})\times \lambda_{l}^{4}\times (\frac{I_{G}}{I_{D}}),$$

where $\lambda_{l}$ is the wavelength of the Raman laser, which equals 532 nm, and I_G_ and I_D_ are intensities of the G and D band peaks in the Raman spectrum of graphene, respectively.

Scanning electron microscopy (SEM) and energy-dispersive X-ray (EDX) analysis were performed using a Phenom ProX scanning electron microscope (Phenom, Eindhoven, The Netherlands). No conductive layer was deposited on the samples and they were analyzed as prepared. For EDX analysis, an acceleration voltage of 15 kV was used.

The phase purity and crystallinity of the synthesized materials were examined using X-ray diffraction (XRD) with a Riguku Ultima IV diffractometer (Tokyo, Japan). The X-ray beam was nickel-filtered Cu*Kα*_1_ radiation (*λ* = 0.1540 nm) operating at 40 kV and 40 mA. XRD data were collected from 3 to 50° (2*q*) at a scanning rate of 2° min^−1^, with a step size of 0.02°. To provide high-intensity, high-resolution measurements, parallel beam geometry and the D/teX Ultra, a high-speed one-dimensional X-ray detector, were used.

Water contact angles were measured using the method suggested by Zisman [46], employing an optical goniometer with a digital camera installed in the axial extension of its lens. Results are presented as mean values of three replicates.

Nanoindentation measurements were made using an Agilent G200 (Santa Clara, CA, USA) instrument with a load force of 30 nN, depth control set to 45 μm, and a Poisson ratio of 0.49. For each material, 100 measurements were taken in a 10 × 10 rectangular array on different spots on the surface of the material samples.

The sheet resistances of the materials were measured using a four-point probe meter A&M Fell Ltd. (London, UK) with golden plated needles as contacts, Keithley 224 (Solon, OH, USA) as a current source, and the digital multimeter Keysight 34461A (Santa Rosa, CA, USA). Calculation of the sheet resistance was carried out using the following Equation (2) [47]:

(2) $$R=4.53\cdot \frac{U}{I}\cdot k$$

where R is the sheet resistance (Ω/sq); U is measured voltage; I is the current; k is a geometric factor (0.85) [47].

Thermogravimetric analysis (TGA) was carried out on a TGA Q500 thermogravimetric analyzer (TA Instruments, New Castle, DE, USA) under a nitrogen flow (purity stream of 99.999%) of 60 mL min^−1^ and oxygen (purity stream of 95.90%) flow of 60 mL min^−1^ in the temperature range from 25 to 700 °C at a heating rate of 5 °C min^−1^.

Differential scanning calorimetry (DSC) was performed using a DSC Q1000 calorimeter with an RCS cooling unit (TA Instruments, New Castle, DE, USA). All DSC scans were conducted in the temperature range from −90 to 260 °C in three cycles: the first heating cycle from −90 to 260 °C, the second cooling cycle from 260 to −90 °C and the third heating cycle from −90 to 260 °C, at heating and cooling rates of 10 and 5 °C min^−1^, respectively. The instrument was calibrated using standard metal indium, and all experiments were conducted under a nitrogen flow of 50 mL min^−1^. Material samples, ranging from 9 to 13 mg, were analyzed in crimped aluminum pans (TA Instruments).

Each thermogram (DSC and TGA) was analyzed using TA Advantage Universal Analysis 2000 software.

## 3. Results and Discussion

The composite substrates demonstrated excellent film-forming capabilities, being easily peeled from the molds. As Triton is added to the PDMS matrix, the composites become opaque. The color of the composites transitions from ivory (1 wt.% Triton) to white (30 wt.% Triton) with increasing Triton content. Photographs of LIG/PDMS/Triton materials are depicted in Figure 2b. Laser irradiation resulted in distinct color changes in the materials. The materials changed color to shades of grey, which darkened to black with increasing Triton content (Figure 2b).

The materials were extensively characterized to assess their structural, surface, thermal, mechanical, and conductive properties.

### 3.1. ATR-FTIR Spectroscopy Results

**Figure 3:**
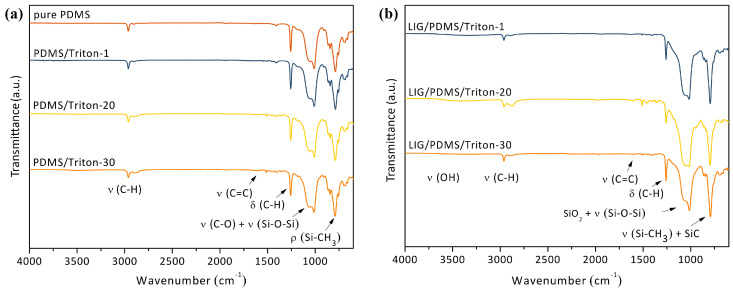
Attenuated total reflection Fourier-transform infrared spectroscopy (ATR-FTIR) spectra of (**a**) PDMS/Triton composites and (**b**) LIG/PDMS/Triton materials.

The ATR-FTIR spectra of pure PDMS, PDMS/Triton, and LIG/PDMS/Triton materials with different contents of Triton are depicted in Figure 3. The pure PDMS network exhibited strong absorption peaks at 2962 and 2905 cm^−1^, associated with the asymmetric and symmetric stretching vibrations of the methyl group, respectively (Figure 3a). Peaks at 1415 and 1259 cm^−1^ corresponded to the asymmetric and symmetric deformations of the methyl group, respectively. Peaks at 1064 and 1113 cm^−1^ were attributed to the asymmetric stretching vibration and asymmetric deformation of Si−O−Si bonds, respectively. A notable peak at 790 cm^−1^ was indicative of CH_3_ rocking and Si-C stretching within the Si-CH_3_ group [34].

The FTIR spectra of the PDMS/Triton composite films displayed the typical peaks of the PDMS elastomer network, along with peaks associated with the presence of Triton X-100, which increased in intensity as Triton content rose from 1 to 30 wt.% (Figure 3). A peak with relatively low intensity was observed at ~1600 cm^−1^, corresponding to aromatic ν_(C=C)_. This new peak was observed in all the PDMS/Triton composite films, but it was absent in pure PDMS. The peak at 1000–1080 cm^−1^, corresponding to ν_(C-O-C)_, overlapped with the ν_(Si-O-Si)_ peak.

After laser irradiation of the PDMS/Triton composite films, the resultant LIG/PDMS/Triton materials were also examined with FTIR spectroscopy, as shown in Figure 3b. Aside from the peaks observed in the materials without graphene, a new peak appears at 3456 cm^−1^, corresponding to -OH groups. The weak absorption peaks corresponding to the C=C stretching vibrations at 1600 cm^−1^ appeared in the LIG/PDMS/Triton materials. The peaks at 1070 and 1015 cm^−1^ in the FTIR spectra of LIG/PDMS/Triton composites were associated both with the ν_(Si-O-Si)_ peak and with silica nanoparticle formation [34]. In LIG/PDMS/Triton materials, the absorption peak at 790 cm^−1^, attributed to the formation of silicon carbide (SiC), increased in intensity compared to the intensities noted in their counterpart PDMS/Triton composite films [34]. We attributed the formation of SiO_2_ nanoparticles and SiC to the thermal degradation of the PDMS matrix in the PDMS/Triton composites.

### 3.2. SEM Analysis

**Figure 4:**
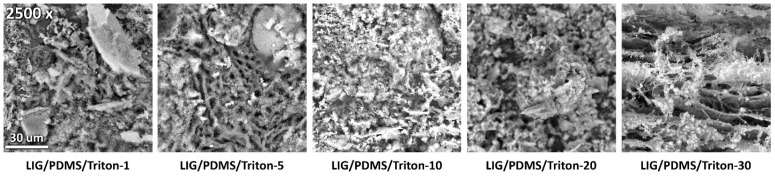
Scanning electron microscope (SEM) images of LIG on PDMS/Triton at magnification 2500×.

**Figure 5:**
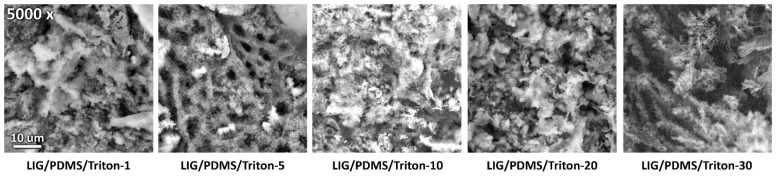
SEM images of LIG on PDMS/Triton at magnification 5000×.

SEM was employed to elucidate the surface morphology of LIG on PDMS/Triton composites. Figure 4, Figure 5, and Figure S1 (Supplementary Material) depict SEM of LIG produced from precursors with different Triton contents in the PDMS matrix, shown at magnifications of 2500× (Figure 4), 5000× (Figure 5), and 10,000× (Figure S1). The micrographs reveal that LIG is a foam-like structure with a micro-scale porous texture within the carbonized framework. The pores occur due to the rapid release of gaseous byproducts during the laser-induced graphenization process [48]. The lateral size of the pores ranged between 3 and 8 μm, as determined with image analysis (Image-Pro Plus version 6.0.0.260). The morphology comprises two primary components: (i) a matrix with laser-induced graphene micropores and (ii) laser-synthesized SiO_2_ nanoparticles and SiC crystallites on the surface of the PDMS matrix. This unique nanostructured SiO_2_ layer formation through direct laser synthesis offers potential for exploration in applications such as microfluidic devices or drug delivery systems [49,50].

The elemental composition of the structures, obtained with energy-dispersive X-ray analysis (EDX) averaged over each micrograph, is depicted in Table 1. Generally, the atomic and weight concentrations of Si increased with rising Triton content in the PDMS matrix (Table 1). Large amounts of Si on the surface of LIG are likely due to the formation of SiO_2_ nanoparticles and SiC crystallites. The concentration of oxygen by weight percent decreases with increasing Triton content. The fact that atomic and weight percent concentrations of carbon, oxygen and silicon do not follow the same trends with increasing Triton content may indicate that chemical bonds change with changing Triton content.

### 3.3. Raman Spectroscopy

**Figure 6:**
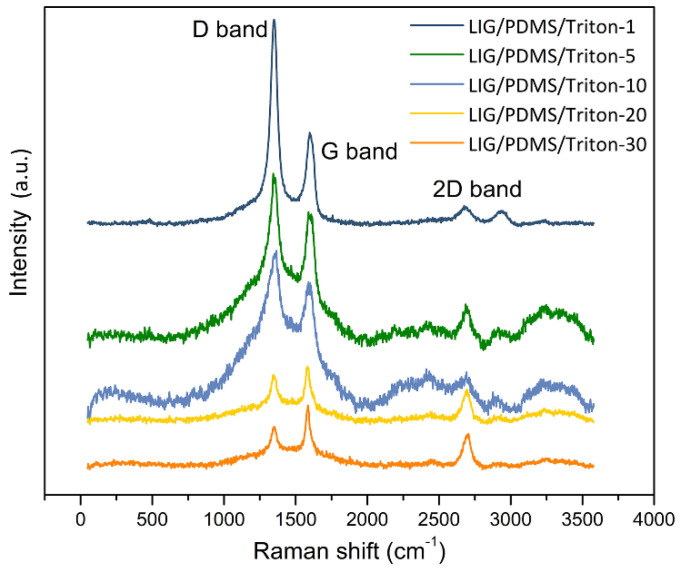
Raman spectra of LIG on PDMS/Triton with different Triton X-100 contents.

Raman spectroscopy provided significant insights into the graphenization process of LIG on PDMS/Triton composite films. The Raman spectrum of LIG on PDMS/Triton composites (Figure 6) exhibited the typical signature of graphene. Analyzing the LIG formed on PDMS/Triton X-100 composites across the different contents of Triton X-100 revealed three distinctive peaks: the D peak at approximately 1350 cm^−1^, which was indicative of defects, vacancies, and bent sp^2^ bonds; the G peak at approximately 1580 cm^−1^, arising from a first-order inelastic scattering process involving the degenerate iTO and iLO phonons at the G point (E2g mode); and the 2D peak at approximately 2700 cm^−1^, resulting from second-order zone boundary phonons [9]. Additionally, a weak signal, i.e., a shoulder associated with the D′ peak at approximately 1630 cm^−1^, indicative of material defectiveness, was observed overlapping partially with the G peak [9]. It is noteworthy that producing LIG on a pure PDMS matrix is unfeasible within the utilized parameter range; this is due to PDMS’s structure that predominantly consists of siloxane chains with a very low carbon content.

Raman spectroscopy is commonly employed to obtain information on the vibrational modes of molecules. However, it has emerged as a valuable technique for estimating diverse material properties, such as the number of graphene layers, crystalline size, and defect concentration. Herein, we present an in-depth Raman analysis.

The deconvolution of the D and G peaks of the Raman spectra illustrated in Figure 6 is presented in Figure S2, while Figure S3 depicts the deconvolution of peaks in the 2D region. We fitted the first-order region to five functions—three Voigt and two Gaussian—alongside fitting the second-order region to two Gaussian functions [51]. Band parameters obtained from this fitting process are shown in Tables S1 and S2.

**Figure 7:**
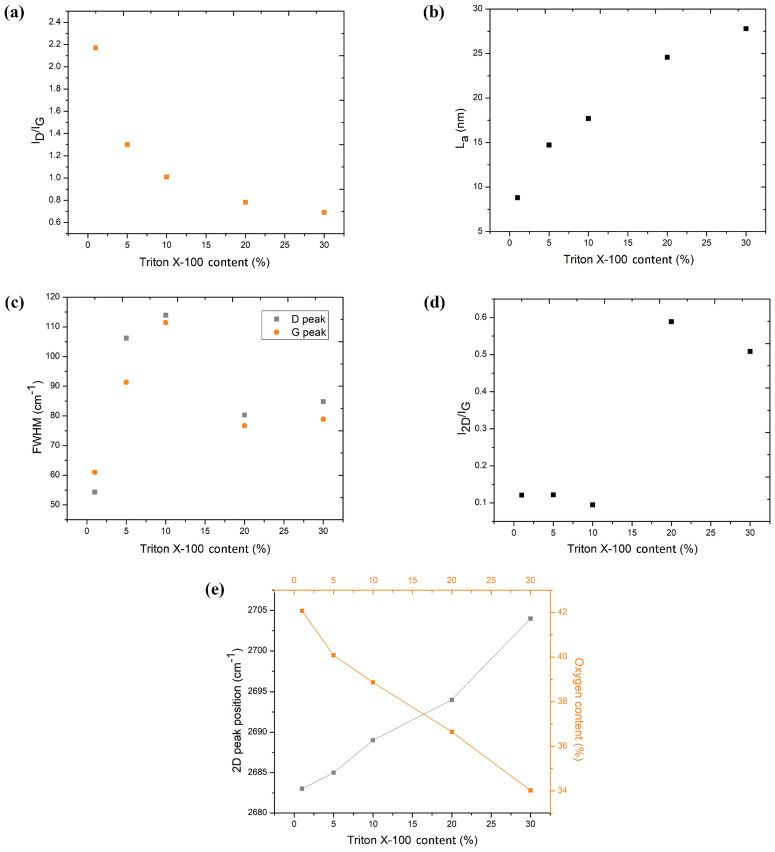
(**a**) Variation in I_D_/I_G_ versus Triton content in the PDMS/Triton composite. (**b**) Dependence of the La with the Triton X-100 content. (**c**) The relationship between the full width at half maximum (FWHM) of the D and G peaks and Triton X-100 content. (**d**) Dependence of the I_2D_/I_G_ ratio with the Triton content. (**e**) Evolution of the position of the 2D peak as a function of Triton content and oxygen content, determined by SEM-EDX analysis (Table 1).

The intensity ratio of the D to G band (I_D_/I_G_) serves as a metric for assessing material disorder [52]. This ratio is an indicator of the degree of defects present in graphitic materials [11]. Notably, as inferred from Figure 7a, the I_D_/I_G_ ratio attains its minimum at the highest Triton X-100 content (Table S3), suggesting the highest quality of LIG at 30 wt.% Triton X-100 content.

Raman spectroscopy also facilitates the estimation of crystallite size (L_a_) of graphitic materials, which is proportional to I_G_/I_D_ [45]. The value of L_a_ with increasing Triton content, obtained from Equation (1), is depicted in Figure 7b. L_a_ increases from 7 to 28 nm as Triton content increases from 1 wt.% to 30 wt.%. Such a high value of L_a_ is indicative of excellent crystallinity. For example, the crystallite size in films of graphene obtained from liquid-phase exfoliation was only on the order of 20 nm [53].

To assess structural order levels among the LIG materials, we analyzed the relationship between the full width at half maximum (FWHM) of the D and G peaks and Triton X-100 content, as shown in Figure 7c. A decrease in the FWHM of the G peak indicated the presence of larger sp^2^ grains, suggesting the formation of better-defined graphene domains at higher Triton content (20 wt.% and 30 wt.%) compared to 5 wt.% and 10 wt.%. The high FWHM values for the G and D peaks at Triton contents of 5 wt.% and 10 wt.% suggest the presence of amorphous or disordered material [39].

Raman spectroscopy also offers insights into the number of layers within graphene domains. In our investigation, the calculated I_2D_/I_G_ ratios were all lower than 1, suggesting the formation of multilayered graphene structures (Figure 7d; Table S3). These results are consistent with those obtained by Raman analysis of LIG formed on other precursor materials [40,54]. In the case of PDMS/Triton composites, the composite materials with contents of Triton of 20 wt.% and 30 wt.% result in graphene with the fewest number of layers.

Previous studies have demonstrated that both doping [55] and strain [56] can induce a shift in the 2D peak position. In our materials, the position of the 2D peak shifts towards longer wavenumbers with increasing Triton content, and with decreasing oxygen content, as depicted in Figure 7e. This shift can be attributed to changes in the oxygen content of LIG as Triton is added to the precursor, or to strain resulting from different thermal expansion coefficients of LIG and the precursor substrate. Given the simultaneous alterations of oxygen content and strain, determining the exact cause of the 2D peak position shift is a challenge.

### 3.4. XRD Analysis

**Figure 8:**
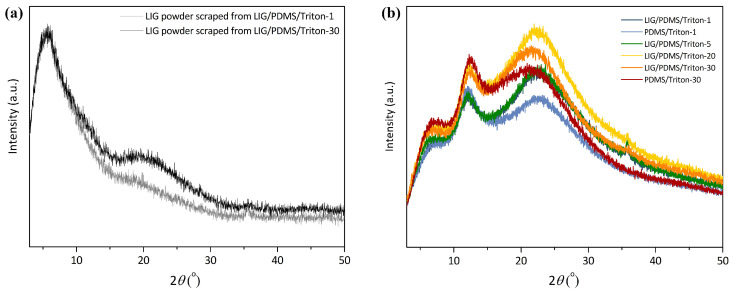
X-ray diffraction (XRD) patterns of the prepared PDMS/Triton composite films and LIG/PDMS/Triton materials. (**a**) LIG powder scraped from LIG/PDMS/Triton materials; (**b**) PDMS/Triton and LIG/PDMS/Triton films.

XRD patterns of PDMS/Triton composites, LIG/PDMS/Triton materials, and powdered LIG scraped from LIG/PDMS/Triton are depicted in Figure 8.

The XRD pattern of powdered LIG scraped from polymer films (Figure 8a) showed a broad peak of weak intensity at 2θ = 20.5° [9,57], characteristic of graphene, and corresponding to the (002) plane of LIG. The LIG produced from a precursor with 30 wt.% Triton exhibits a more pronounced peak than the LIG produced from a precursor with 1 wt.% Triton, which is another indicator that higher contents of Triton are favorable for induction of graphene (Figure S4, Supplementary Materials). The XRD pattern also contains a diffraction peak at 2θ = 36° [57], corresponding to the (111) diffraction planes of crystalline β-SiC, demonstrating the laser-induced transformation from PDMS to SiC. The identification of these peaks was performed using graphene and SiC reference cards from the instrument (Figure S5, Supplementary Materials).

The inter-planar distance (d) was calculated using Bragg’s equation, nλ = 2dsinθ. For the first diffraction order (*n* = 1), x-ray wavelength of 1.54056 Å, and a 2θ angle of 20.5°, the calculated value of the inter-planar distance between (002) planes was 4.329 Å, which is in accordance with the literature data for graphene [9]. For the 2θ angle of 36°, the calculated value of the inter-planar distance between (111) planes was 2.4927 Å [34,58].

XRD patterns of the prepared materials (Figure 8b) showed an amorphous halo at 2θ = 12°, originating from the PDMS phase, and an amorphous halo at 2θ = 22°, originating from the Triton X-100 phase. The positions of the peak related to Triton X-100 and the peak based on graphene overlap. In LIG/PDMS/1 wt.% Triton and LIG/PDMS/30 wt.% Triton, both graphene-containing materials, the peaks at this position are greater in intensity than the peaks in PDMS/1 wt.% Triton and PDMS/30 wt.% Triton (both without graphene). As the Triton content in the PDMS matrix increases, the peak at 2θ = 22°, originating from the Triton phase in the polymer matrix, also increases.

### 3.5. Water Contact Angle Results

**Figure 9:**
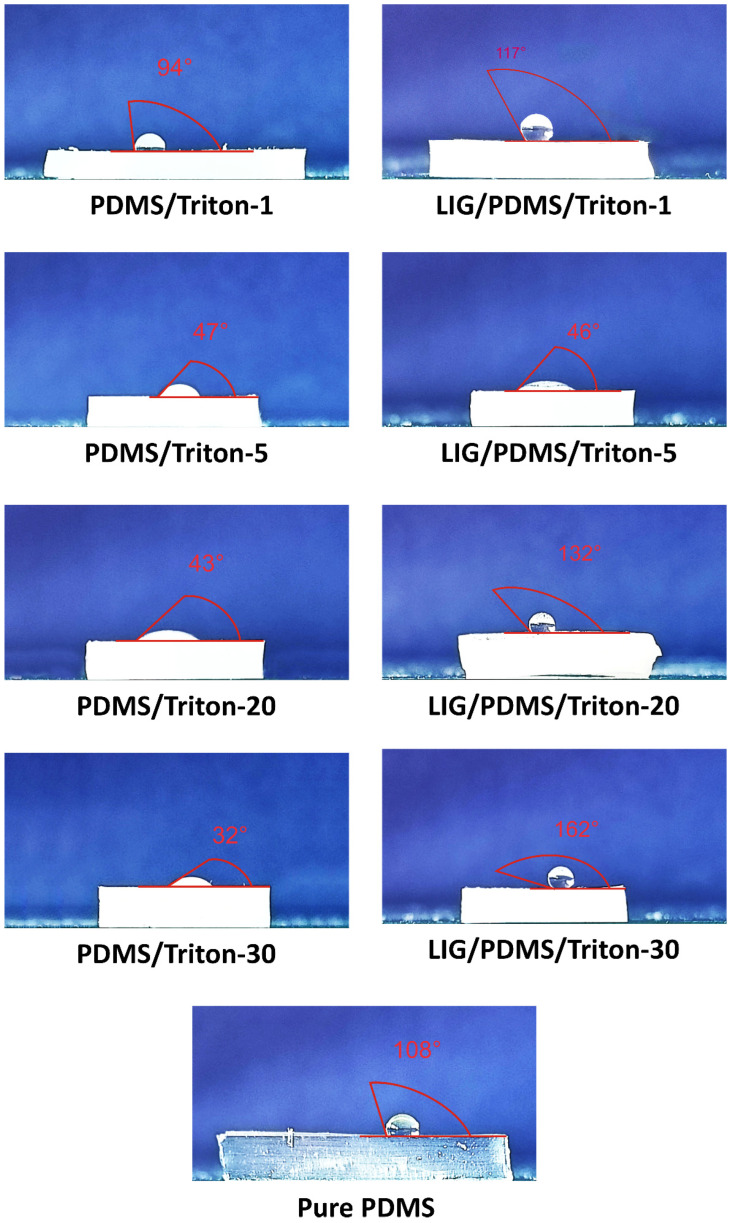
Water contact angles of PDMS/Triton composite films and LIG/PDMS/Triton materials.

The surface characteristics of selected PDMS/Triton composite films and LIG/PDMS/Triton materials were evaluated by measuring the static water contact angle, with particular emphasis on the wettability and hydrophobicity of the materials. A water contact angle of 90° or more indicates a non-wetting surface, i.e., a hydrophobic one, and an angle of more than 150° indicates a superhydrophobic surface. The water contact angle measurements for the PDMS/Triton films and LIG/PDMS/Triton materials are shown in Figure 9. Water contact angles for PDMS/Triton films decreased from 94° to 32° with increasing content of Triton X-100 in the PDMS matrix, whereas the water contact angle for pure PDMS was 108°. This trend is attributed to the migration of PDMS segments to the material surface, driven by the low surface energy of PDMS, which reduces surface tension and covers most of the material’s surface [59,60]. Conversely, as the Triton content decreased, the hydrophobicity of the films increased. High Triton content resulted in smaller water contact angles, suggesting the presence of a significantly hydrophilic surface at the air interface, indicating that the surface wettability of PDMS/Triton is predominantly governed by its chemical composition. PDMS/Triton with 30 wt.% Triton X-100 exhibited the most hydrophilic surface among the synthesized polymer films.

For LIG/PDMS/Triton composite materials, water contact angles varied between 117° and 162°, with materials containing 20 and 30 wt.% Triton exhibiting water contact angles of 132° and 162°, respectively, reflecting their hydrophobic and superhydrophobic nature [61]. The greater hydrophobicity of LIG/PDMS/Triton materials compared to PDMS/Triton composites highlights the influence of surface morphology, enhanced by the presence of LIG on the hydrophobicity of the material surface.

These results provide crucial insights into the material properties influenced by compositional differences in the PDMS/Triton composites and their modifications with LIG, suggesting potential applications where surface-wetting properties are critical.

### 3.6. Nanoindentation Analysis

Nanoindentation is a technique of critical importance for determining the mechanical properties of superficial layers of materials, offering insights that are not readily obtainable through traditional tensile testing methods [59]. This technique was employed to evaluate the nanomechanical properties, specifically the modulus of elasticity (Young’s modulus), hardness, and plasticity, of LIG/PDMS/Triton composite materials. Using our instrumentation, we could measure these parameters only for LIG/PDMS/Triton materials with 20 and 30 wt.% Triton. This was due to the good quality and hardness of LIG on the surface of these materials as compared to the other prepared LIG/PDMS/Triton materials that had less Triton content and a softer PDMS/Triton composite, and also as compared to pure PDMS.

The results, summarized in Table 2, indicate that the Young’s modulus of LIG/PDMS/Triton materials increased with the content of Triton, from 0.239 GPa in LIG/PDMS/20 wt.% Triton to 0.329 GPa in LIG/PDMS/30 wt.% Triton, indicating a stiffening of the PDMS matrix with Triton incorporation. For both materials, the Young’s modulus remained well below a gigapascal, confirming that the synthesized materials have the flexibility and stretchability that is required for applications in wearable electronic devices. The hardness value was the same for both LIG/PDMS/20 wt.% Triton and LIG/PDMS/30 wt.% Triton materials (Table 2).

The plasticity (also called ductility) index reflects a material’s ability to undergo plastic deformation under indentation and correlates with its fracture toughness. The plasticity index was higher in LIG/PDMS/30 wt.% Triton than in LIG/PDMS/20 wt.% Triton, suggesting greater toughness with higher Triton content (Table 2).

### 3.7. Electrical Measurements

**Figure 10:**
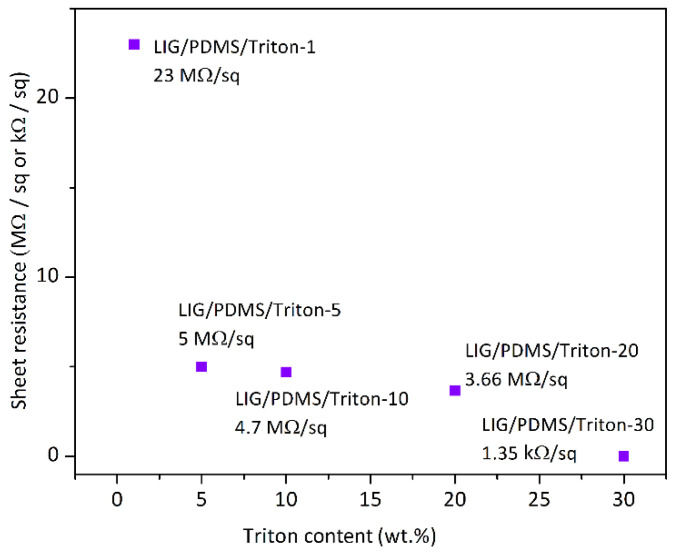
Sheet resistance values of the prepared LIG/PDMS/Triton materials with different Triton X-100 contents.

The four-point probe sheet resistance of the LIG induced on the materials with different Triton contents is shown in Figure 10. Sheet resistance decreased as the Triton content in the PDMS matrix increased. Specifically, the resistance decreases from a high of 23.00 MΩ/sq in LIG/PDMS/1 wt.% Triton to 1.35 kΩ/sq in LIG/PDMS/30 wt.% Triton, which is a reduction of more than four orders of magnitude. This reduction in resistance with increased Triton content is attributed to an increased content of carbon atoms and the presence of aromatic structures within the materials containing Triton, which enhance the conductivity of the LIG layers.

For context, LIG induced on polyimide, a commonly used polymer, typically exhibits sheet resistances around 50 Ω/sq, while the sheet resistance of PDMS/TEG with 30 wt.% TEG in the PDMS matrix was 158.5 Ω/sq [34], highlighting the significant influence of the substrate on the final material’s electrical properties. While our material, LIG/PDMS/30 wt.% Triton, exhibited an order of magnitude higher sheet resistance than the TEG-based materials [34], LIG/PDMS/30 wt.% Triton could still be useful in applications where moderate conductivity suffices or in scenarios where other attributes, such as flexibility and stiffness take precedence, for example in wearable sensor applications. Moreover, the flexibility of PDMS-based materials, combined with their controllable resistance properties, presents a compelling opportunity for implementation in the realm of wearable electronic devices intended for skin attachment.

### 3.8. Thermal Characterization

#### 3.8.1. TGA Results in Nitrogen

**Figure 11:**
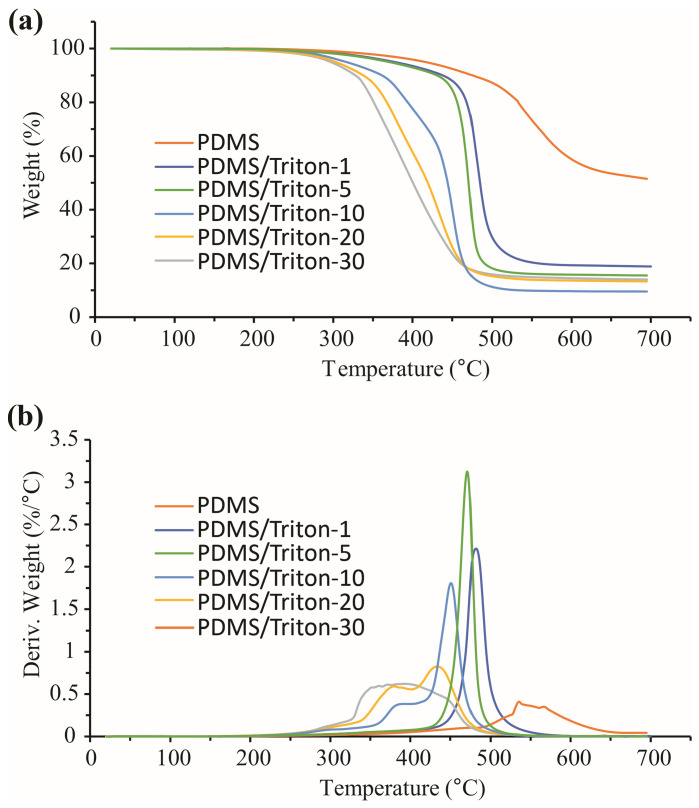
Thermal degradation of PDMS/Triton composite films with different contents of Triton (1–30 wt.%) analyzed in a nitrogen atmosphere. (**a**) thermogravimetric analysis (TGA) curves. (**b**) derivative thermogravimetric analysis (dTG) curves.

The thermal degradation of the PDMS/Triton composite films and LIG/PDMS/Triton materials in two different gas atmospheres, nitrogen, and oxygen, was studied. The nitrogen atmosphere TGA and dTG curves for the PDMS-based materials (without LIG) are shown in Figure 11a,b, respectively. In Table 3a,b, the onset temperatures of the beginning of the thermal degradation (T_on_) and total mass losses of the PDMS/Triton composite films and LIG/PDMS/Triton materials are presented.

It can be seen from the TGA/dTG curves (Figure 11, Table 3) that PDMS was the most resistant of this group of materials toward thermal degradation. PDMS started to thermally degrade at 488 °C, while the PDMS/Triton 1–30 wt.% materials started to thermally degrade at lower temperatures (Table 3a). Moreover, the onset temperature for thermal degradation was generally lower with increasing Triton content. Also, the PDMS/Triton composite films were thermally destabilized in terms of the total mass loss (Figure 11, Table 3a), and total mass loss was greater with Triton addition as compared to pure PDMS. The thermal degradation of the powdered LIG (scraped from LIG/PDMS/30 wt.% Triton) in N_2_ began at T_on_ = 439 °C (Table 3b). Based on this onset temperature of thermal degradation (T_on_), the powdered LIG has higher thermal stability compared to the other materials, except in the case of LIG/PDMS/5wt.% Triton (Table 3b). However, considering the total mass loss, LIG’s thermal stability is considerably high, with only a 9.22% total mass loss in N_2_ (Table 3b).

Thermal degradation in all materials occurred in one step, except in the case of higher Triton content (20 and 30 wt.%), when thermal degradation became complex due to the presence of the Triton component.

**Figure 12:**
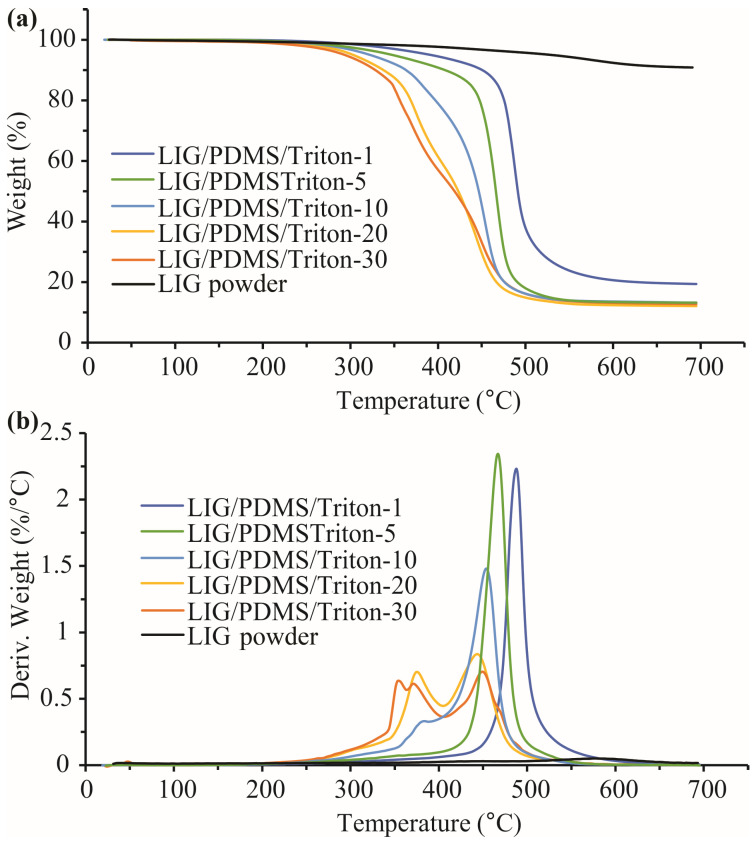
Thermal degradation of LIG/PDMS/Triton materials with different contents of Triton (1–30 wt.%) analyzed in a nitrogen atmosphere. (**a**) TGA curves. (**b**) dTG curves.

TGA was performed on materials with LIG induced, with similar results (Figure 12, Table 3b). Since the LIG is only several micrometers thick, on top of materials that are several millimeters thick, the influence of LIG on the overall material thermal stability is negligible.

#### 3.8.2. TGA Results in Oxygen

The thermal stability of PDMS/Triton composite films and LIG/PDMS/Triton materials in the presence of oxygen was also studied.

**Figure 13:**
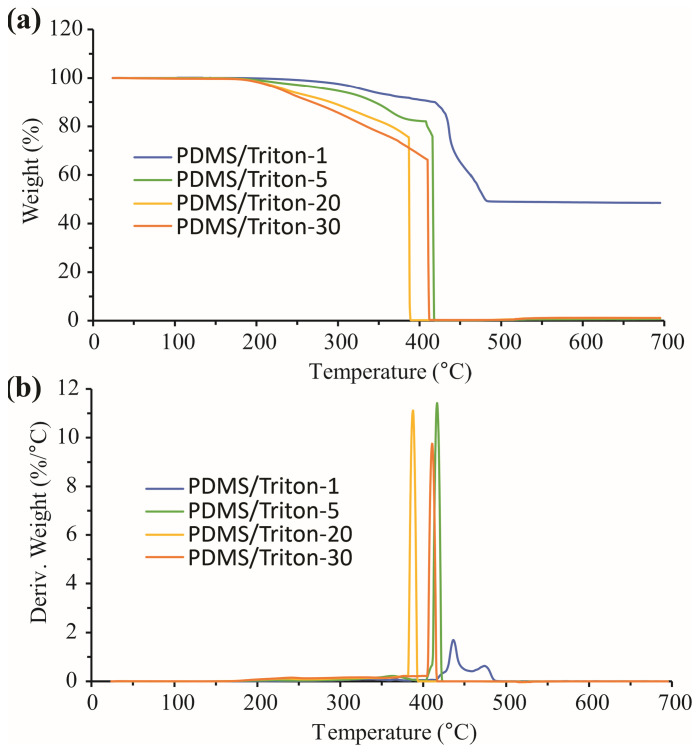
Thermal degradation of PDMS/Triton composite films with different contents of Triton (1–30 wt.%) analyzed in an oxygen atmosphere. (**a**) TGA curves. (**b**) dTG curves.

It can be seen from the TGA and dTG curves (Figure 13, Table 4a) that PDMS/1 wt.%, 5 wt.% and 10 wt.% Triton were the most resistant of the examined PDMS-based films towards thermal degradation, which started at about 315 °C in those films. The thermal degradation temperature of PDMS/Triton films (20 wt.% and 30 wt.%) in oxygen decreased with increasing Triton content (Figure 13, Table 4a).

Thermal degradation in all these PDMS-based materials took place in one step, although at contents of Triton 1 wt.%, this one-step mass loss became more complex.

**Figure 14:**
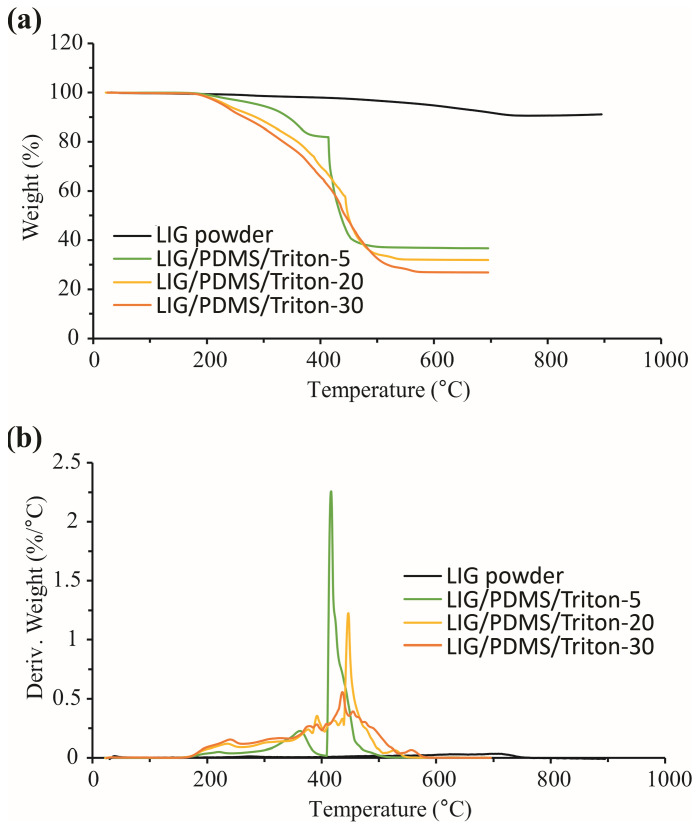
Thermal degradation of LIG/PDMS/Triton materials with different contents of Triton (1–30 wt.%) analyzed in an oxygen atmosphere. (**a**) TGA curves. (**b**) dTG curves.

The TGA and dTG curves (Figure 14, Table 4b) for the materials containing LIG show similar thermal stability as counterpart materials without LIG according to the thermal degradation onset temperatures (T_on_) under oxygen (Figure 14, Table 4b). In the materials with LIG (Figure 14), under oxygen, one-step mass loss was detected, and that one-step loss became more complex with increasing Triton content (similar to the situation with nitrogen). The TGA and dTG curves showed pure LIG, i.e., powdered LIG was the most thermostable of this group of materials according to the thermal degradation onset temperature (519 °C) and the total mass loss (9.44%) (Table 4b).

Generally, from the TGA and dTG results obtained, the PDMS/Triton composite films were more thermostable in nitrogen than in oxygen, and the production of LIG on their surfaces did not influence the thermal stability of the materials in both nitrogen (Table 3) and oxygen (Table 4) atmospheres.

The difference in the thermal stability of PDMS in nitrogen and oxygen is a consequence of different degradation mechanisms in these two atmospheres [62]. In an inert atmosphere (ours was nitrogen), PDMS degradation takes place thanks to the redistribution of siloxane bonds, leading to the formation of thermodynamically stable cyclic products, while at elevated temperatures in the presence of oxygen, oxidation of C-H bonds occurs, which weakens Si-C bonds and causes intermolecular cross-linking and other degradation processes with the formation of SiO_2_ [62]. In an oxygen atmosphere, the LIG/PDMS/Triton materials have greater thermal stability compared to their counterparts without LIG, which is evident from a lower total mass loss (Table 4a,b). The mechanism of thermal stabilization with LIG could be related to the formation of oxidized LIG [63] in the oxygen atmosphere. It was shown earlier that the addition of graphene oxide to a polymer matrix increases the thermal stability of the matrix [64].

Results of TGA for powdered LIG, obtained by scraping from PDMS/30 wt.% Triton samples, are presented in Supplementary Materials (Figure S6) and compared to the literature data [65].

#### 3.8.3. DSC Results

**Figure 15:**
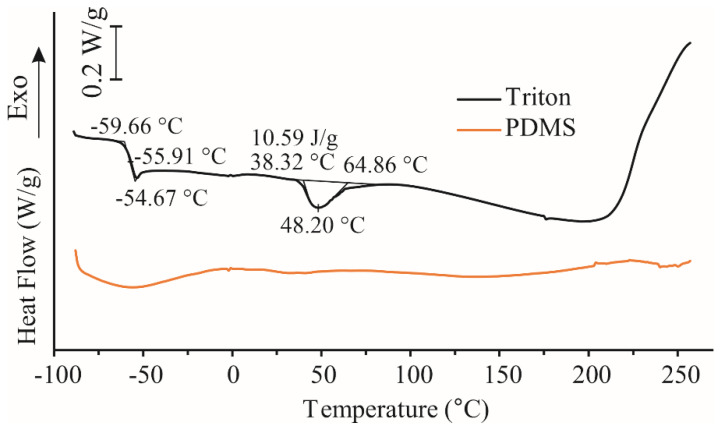
Differential scanning calorimetry (DSC) curves of PDMS and Triton X-100 in a nitrogen atmosphere during the second heating scan.

Figure 15 shows the DSC curves of PDMS and Triton X-100. The glass transition temperature obtained for Triton X is −55 °C (Figure 15). The PDMS DSC curve shows no visible thermal events in the studied temperature range from −90 to 260 °C, which is expected, as for linear PDMS of low molecular weights, the glass transition temperature, T_g_, ranges between −137 °C and −127 °C [66]. The DSC curve for the PDMS we used in our study was similar to the DSC curve of PDMS-20, as reported by Klonos et al. [66]. DSC curves observed in the literature [66] showed that during heating, cold crystallization was barely visible but nonetheless observable at around −85 °C. Cold crystallization was followed by weak single-peaked melting at T_m_~−85 °C, suggesting that probably low-quality crystals had formed, possibly of low density and size [66]. In our study, this transition was not found, as we used different types of sample pans and a different instrument that was capable of scanning in a broad temperature range from −90 to 400 °C, so thermal events outside that range could not be observed.

The DSC curve of Triton X-100 was more complex. A glass transition was evident at −55 °C, and a broad endotherm with a peak at 48 °C (a second heating scan is also shown in Figure 15). Those results are in agreement with results from the literature [67]. Merino et al. [67] found that under specific conditions, it is possible to enlarge the temperature range where Triton X-100 exhibits both liquid and supercooled liquid properties, with vitrification occurring only around −67 °C; this ability could be advantageously used in different applications as a means of cryopreservation in biological systems. The broad endotherm with a peak at 48 °C is related to phase transitions of the binary Triton X-100/water system [68].

Another characteristic of Triton X-100 behavior as reported by Klonos et al. [66] was also found in our study, i.e., in the second heating scan, the cold crystallization peak disappeared completely, while T_g_ at −55 °C became more pronounced. It can be proposed that Triton becomes more amorphous after the first heating and cooling scan, under the given temperature conditions (−90 to 250 °C and a heating rate of 5 °C min^−1^).

**Figure 16:**
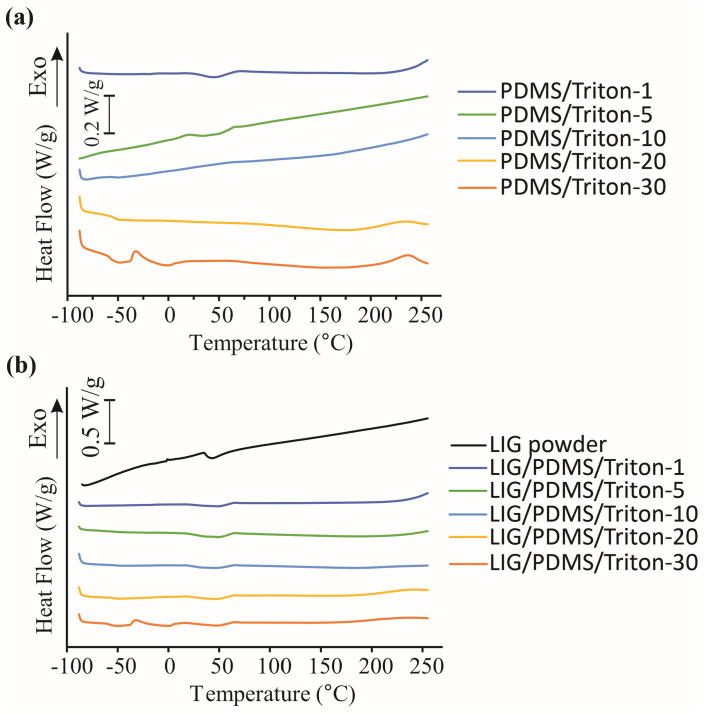
(**a**) DSC curves of PDMS/Triton composite films. (**b**) DSC curves of LIG/PDMS/Triton materials (the second heating scan).

The PDMS/Triton composite films obtained in this study, with different contents of Triton X-100 in PDMS (1–30 wt.%), were analyzed by DSC. The results of DSC obtained during the first and second heating scans are presented in Tables S4–S7 (Supplementary Materials). The DSC curves of the PDMS/Triton composites, containing 10 wt.% or more Triton, during the first heating scan (Figure 16a and Figure S7; Table S4), have the Triton glass transition (T_g_) between −48 °C and −58 °C, an exothermal peak at around −31 °C attributed to the Triton cold crystallization and endothermal peak with T_m_ between 0.4 °C and 1.5 °C representing Triton melting.

The DSC curves of the second heating scan of the PDMS/Triton composites are presented in Figure 16a. The DSC curves of the composites with low contents of Triton (1 wt.% and 5 wt.%) exhibited almost the same thermal behavior. The DSC curves of the PDMS/Triton composites, (second heating scan) containing 10 wt.% (Figure 16a and Figure S8; Table S5) or more Triton (Figure 16a; Table S5) a glass transition (T_g_)_,_ between −35 °C and −58 °C, appeared, but a Triton melting endotherms are missing. The DSC curve of the composite containing the 30 wt.% of Triton (Figure 16a), on the second heating scan, has the Triton cold crystallization exotherm at around −31 °C and Triton melting endotherm with temperature maximum T_m_ about −1.5 °C [67].

We propose that at the Triton content of 30 wt.%, there was an overload of Triton, so the excess was external to the polymer matrix and expressed its own thermal behavior.

The LIG/PDMS/Triton materials in our study, with different contents of Triton X-100 (1–30 wt.%) in PDMS, were also analyzed with DSC (Figure 16b; Tables S6 and S7). The thermal behaviors of the materials with 1 wt.% to 20 wt.% Triton with LIG were very similar to the thermal behaviors of their counterpart materials without LIG. Moreover, the behavior of Triton in the LIG material with 30 wt.% Triton was the same as in the counterpart polymer material without LIG. Overall, the presence of LIG on the PDMS/Triton composites did not influence the materials’ thermal behavior (determined by DSC) in the temperature range between −90 and 260 °C.

The DSC curve of powdered LIG (scraped from LIG/PDMS/30 wt.% Triton) showed one endothermic transition peak at about 48 °C, which was assigned as most likely being the conformational and/or phase transition of the material, as water loss was excluded on the second heating cycle (Figure 16b).

## 4. Conclusions

This research successfully demonstrates the production of LIG on novel cross-linked PDMS/Triton X-100 precursor substrates, marking a significant advancement in the field of flexible electronic materials. Using a CO_2_ laser, graphene was efficiently produced in a single step across substrates with various Triton X-100 content (1–30 wt.%) embedded within the PDMS matrix. Systematic characterization revealed that the physicochemical, mechanical, and electrical properties are tunable and influenced by Triton content, and this latter factor is crucial for controlling the graphenization process. Specifically, the increase in the I_2D_/I_G_ ratio and the decrease in the I_D_/I_G_ ratio with higher content of Triton indicates fewer graphene layers with fewer defects as Triton is added. The addition of LIG on PDMS/Triton materials creates a superhydrophobic surface compared to the counterpart materials without graphene. Stiffness, fracture toughness and hydrophobicity of LIG/PDMS/Triton materials increase with increasing Triton content. Electrical measurements show decreased sheet resistance with higher Triton content, suggesting the possibility of tailored conductivity for specific applications. Thermal analyses highlighted the materials’ stability and decomposition patterns, essential for device integration under various conditions. Thermal analysis under different atmospheric conditions shows that Triton content affects the materials’ thermal stability and degradation characteristics. PDMS with 30 wt.% Triton is optimal for high-quality LIG, combining ease of processability with beneficial functional properties. These promising results pave the way for using LIG on PDMS/Triton composite substrates in advanced technological applications, particularly wearable flexible electronics, such as sensors, based on the balance of mechanical flexibility, electrical conductivity, and ease of fabrication. The scalable, cost-effective synthesis approach and potential integration with other conductive polymers offer prospects for mass production of flexible electronic devices. This research advances the scientific understanding of graphene-based flexible materials and sets a foundation for future explorations into other elastomeric substrates, potentially revolutionizing next-generation electronic device design and fabrication.

## Figures and Tables

**Table 1:** Elemental composition of the materials produced, determined using electron-dispersive X-ray spectroscopy (EDX).

Material	Atomic Percent			Weight Percent		
	C	O	Si	C	O	Si
LIG/PDMS/5 wt.% Triton	54.2	37.2	8.6	43.7	40.1	16.2
LIG/PDMS/10 wt.% Triton	46.4	41.1	12.5	40.9	38.9	20.2
LIG/PDMS/20 wt.% Triton	44.6	40.5	14.9	39.8	36.6	23.6
LIG/PDMS/30 wt.% Triton	45.1	37.4	17.5	37.7	34.0	28.3

**Table 2:** Surface mechanical properties of LIG/PDMS/Triton materials.

Material	Young’s Modulus/GPa	Hardness/GPa	Plasticity (Ductility) Index/GPa
LIG/PDMS/20 wt.% Triton	0.239 ± 0.089	0.001	239
LIG/PDMS/30 wt.% Triton	0.329 ± 0.072	0.001	329

**Table 3:** Onset temperatures of the beginning of the thermal degradation (T_on_) and total mass losses of the (**a**) PDMS/Triton composites and (**b**) LIG/PDMS/Triton materials, in a nitrogen atmosphere.

**(a)**		
**Material**	**T_on_/°C**	**Total Mass Loss/%**
Pure PDMS	488.10	48.52
PDMS/5 wt.% Triton	463.30	81.41
PDMS/10 wt.%Triton	418.10	90.44
PDMS/20 wt.% Triton	354.40	86.67
PDMS/30 wt.% Triton	324.30	85.90
**(b)**		
**Material**	**T_on_/°C**	**Total Mass Loss/%**
LIG	439.30	9.22
LIG/PDMS/5 wt.% Triton	446.30	86.82
LIG/PDMS/10 wt.% Triton	395.50	86.82
LIG/PDMS/20 wt.% Triton	342.40	87.94
LIG/PDMS/30 wt.% Triton	327.20	87.27

**Table 4:** Onset temperatures of the beginning of the thermal degradation (T_on_) and total mass losses of the (**a**) PDMS/Triton composites and (**b**) LIG/PDMS/Triton materials, in an oxygen atmosphere.

**(a)**		
**Material**	**T_on_/°C**	**Total Mass Loss/%**
Pure PDMS	317.10	62.26
PDMS/5 wt.% Triton	315.13	99.37
PDMS/10 wt.%Triton	315.30	99.37
PDMS/20 wt.% Triton	315.20	99.20
PDMS/30 wt.% Triton	194.80	89.91
**(b)**		
**Material**	**T_on_/°C**	**Total Mass Loss/%**
LIG	519.44	9.44
LIG/PDMS/5 wt.% Triton	295.69	99.35
LIG/PDMS/10 wt.% Triton	324.10	63.32
LIG/PDMS/20 wt.% Triton	323.50	63.40
LIG/PDMS/30 wt.% Triton	194.28	68.11

## Data Availability

Data is contained within the article or Supplementary Material.

## Supplementary Materials

The following supporting information can be downloaded at: https://www.mdpi.com/article/10.3390/polym16223157/s1, Raman deconvolution analysis of D, G and 2D bands; SEM images of LIG on PDMS/Triton at 10,000× magnification; XRD patterns of graphene and SiC from instrument data; TGA and dTG curves of the LIG powder scraped from LIG/PDMS/30 wt.% Triton in both a nitrogen and an oxygen atmosphere; DSC curves of PDMS/10 wt.% Triton and LIG/PDMS/10 wt.% Triton with T_g_ and T_m_ of the thermal transitions during the first and second heating scans; DSC results for the prepared materials. Figure S1: SEM images of LIG on PDMS/Triton with different Triton content (1-30 wt.%) at magnification 10,000×; Figure S2: Deconvolution of D and G bands for the Raman spectra depicted in Figure 4. (a) LIG on PDMS/1 wt.% Triton, (b) LIG on PDMS/5 wt.% Triton, (c) LIG on PDMS/10 wt.% Triton, (d) LIG on PDMS/20 wt.% Triton, (e) LIG on PDMS/30 wt.% Triton; Table S1: Parameters obtained from the deconvolution of D and G bands for the spectra depicted in Figure S2; Figure S3: Deconvolution of the 2D region for the Raman spectra depicted in Figure 4. (a) LIG on PDMS/1 wt.% Triton, (b) LIG on PDMS/5 wt.% Triton, (c) LIG on PDMS/10 wt.% Triton, (d) LIG on PDMS/20 wt.% Triton, (e) LIG on PDMS/30 wt.% Triton; Table S2: Parameters obtained from the deconvolution of the 2D region for the spectra depicted in Figure S3; Table S3: I_D_/I_G_ and I_2D_/I_G_ calculated from Raman spectra of LIG/PDMS/Triton materials; Figure S4: XRD patterns of selected PDMS/Triton and LIG/PDMS/Triton materials; Figure S5: XRD patterns of graphene and SiC cards from instrument data; Figure S6: (a) TGA and (b) dTG curves of LIG in nitrogen and oxygen atmospheres; Figure S7: DSC curves of PDMS/Triton-10 and LIG/PDMS/Triton-10 materials with glass transition (T_g_) and temperature maximum (T_m_) of the thermal transitions (the first heating scan); Figure S8: DSC curves of PDMS/Triton-10 and LIG/PDMS/Triton-10 materials with glass transition (T_g_) and temperature maximum (T_m_) of the thermal transitions (the second heating scan); Table S4: DSC results of PDMS/Triton composite films (first heating scan); Table S5: DSC results of PDMS/Triton composite films (second heating scan); Table S6: DSC results of LIG/PDMS/Triton materials (first heating scan); Table S7: DSC results of LIG/PDMS/Triton materials (second heating scan); Ref. [65] is cited in the Supplementary Materials.
