# Supplementary material for: Laser-Induced Graphene on Novel Crosslinked Poly(dimethylsiloxane)/Triton X-100 Composites for Improving Mechanical, Electrical and Hydrophobic Properties
Source: Polymers (Basel). 2024 Nov 13;16(22):3157. doi: 10.3390/polym16223157 (PMC11598474; doi:10.3390/polym16223157)
Supplement: Supplementary file 1 [file polymers-16-03157-s001.zip › polymers-3307663-supplementary.pdf]

Supplementary Information

Laser-induced graphene on novel crosslinked poly(dimethylsiloxane)/Triton X-100 composites for improving mechanical, electrical and hydrophobic properties

Marija V. Pergal [1*], Milena Rašljić Rafajilović [1], Teodora Vićentić [1], Igor Pašti [2], Sanja Ostojić [3], Danica Bajuk-Bogdanović [2], Marko Spasenović [1]

[1] Center for Microelectronic Technologies, Institute of Chemistry, Technology and Metallurgy, University of Belgrade, National Institute of the Republic of Serbia, Njegoševa 12, 11000 Belgrade, Serbia
[2] University of Belgrade - Faculty for Physical Chemistry, Studentski trg 12-16, 11158 Belgrade, Serbia
[3] The Institute of General and Physical Chemistry Studentski trg 12/V, 11158 Beograd, Serbia

*Correspondence: *marija.pergal@ihtm.bg.ac.rs; marijav@chem.bg.ac.rs*

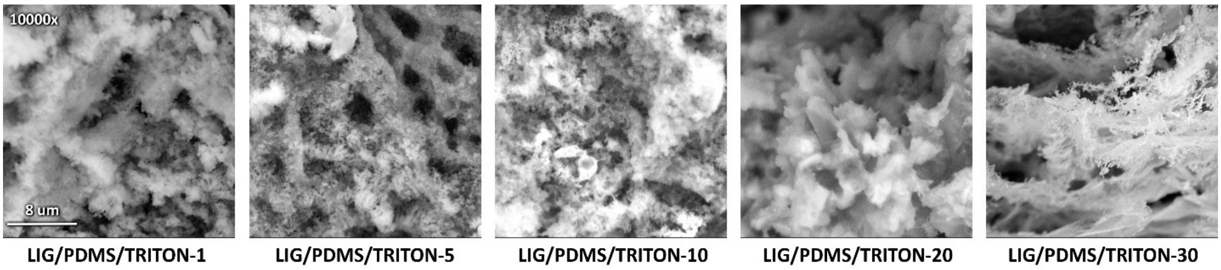

Figure S1. SEM images of LIG on PDMS/Triton with different Triton content (1-30 wt.%) at magnification 10000×.

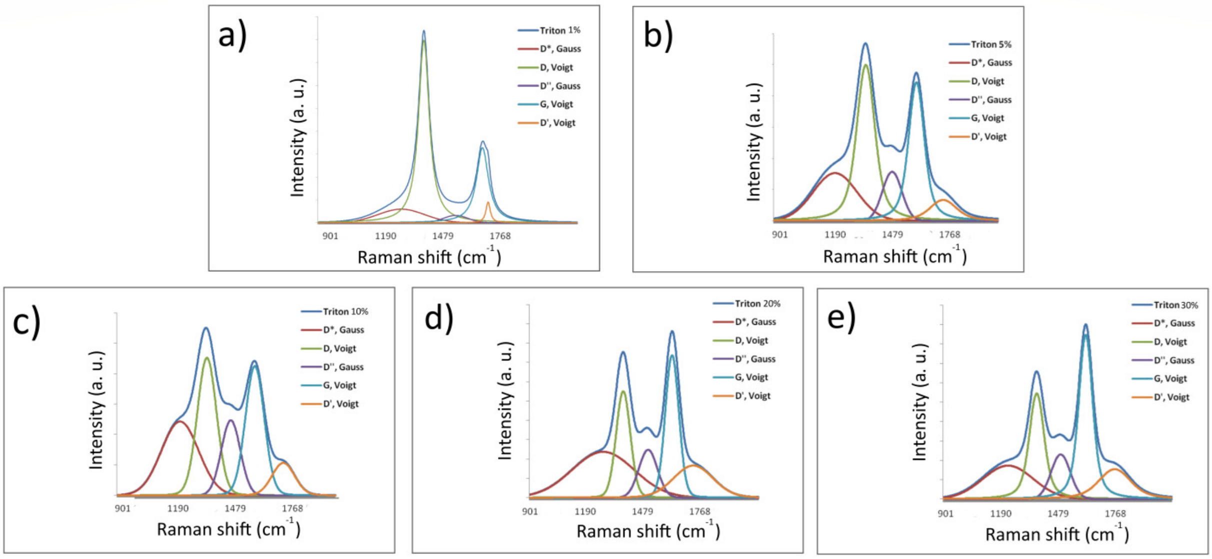

Figure S2. Deconvolution of D and G bands for the Raman spectra depicted in Figure 4. (a) LIG on PDMS/1 wt.% Triton, (b) LIG on PDMS/5 wt.% Triton, (c) LIG on PDMS/10 wt.% Triton, (d) LIG on PDMS/20 wt.% Triton, (e) LIG on PDMS/30 wt.% Triton.

Table S1. Parameters obtained from the deconvolution of D and G bands for the spectra depicted in Figure S2.

	**D***		**D**		**D″**		**G**		**D′**	
	ṽ (cm^{-1})	Area (%)	ṽ (cm^{-1})	Area (%)	ṽ (cm^{-1})	Area (%)	ṽ (cm^{-1})	Area (%)	ṽ (cm^{-1})	Area (%)
**a**	1253	12.4	1352	55.8	1483	3.5	1591	25.6	1619	2.7
**b**	1201	21.4	1354	35.1	1480	9.8	1595	26.9	1713	6.8
**c**	1217	27.1	1353	26.7	1472	14.1	1593	24.6	1735	7.5
**d**	1257	32.9	1350	18.2	1469	10.4	1584	23.3	1682	15.2
**e**	1213	20.3	1352	22.8	1465	10.5	1582	33.0	1707	13.4

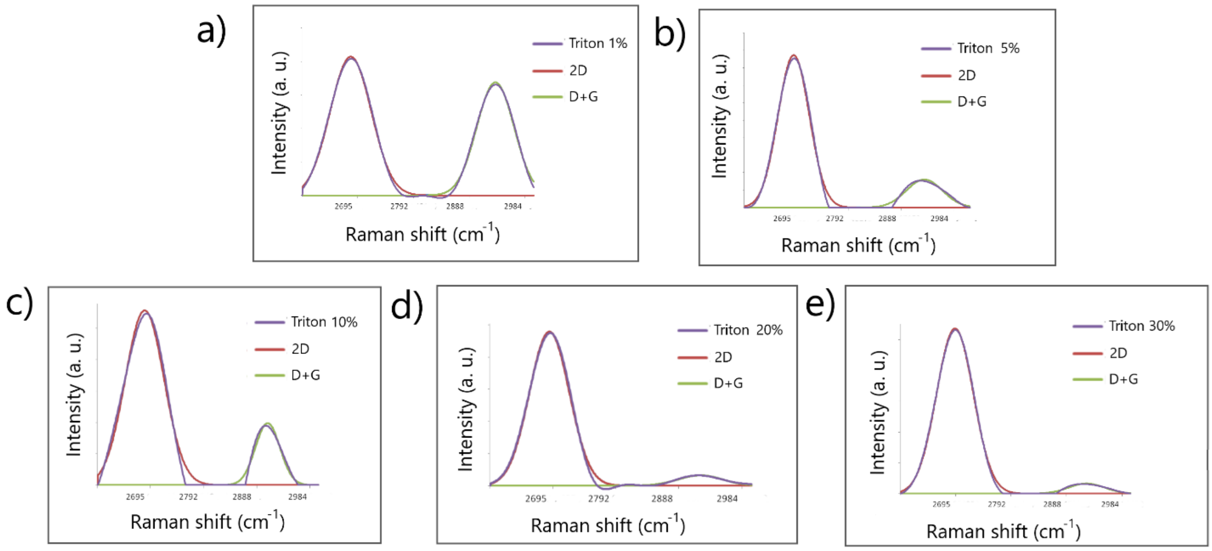

Figure S3. Deconvolution of the 2D region for the Raman spectra depicted in Figure 4. (a) LIG on PDMS/1 wt.% Triton, (b) LIG on PDMS/5 wt.% Triton, (c) LIG on PDMS/10 wt.% Triton, (d) LIG on PDMS/20 wt.% Triton, (e) LIG on PDMS/30 wt.% Triton.

Table S2. Parameters obtained from the deconvolution of the 2D region for the spectra depicted in Figure S3.

	**2D**		**D + G**	
	ṽ (cm^{-1})	Area (%)	ṽ (cm^{-1})	Area (%)
**a**	2684	57.8	2932	42.2
**b**	2690	82.5	2930	17.5
**c**	2685	81.6	2908	18.4
**d**	2690	93.1	2919	6.9
**e**	2694	94.5	2923	5.5

Table S3. I_D/I_G and I_{2D}/I_G calculated from Raman spectra of LIG/PDMS/Triton materials.

**Material**	**I_D/I_G**	**I_{2D}/I_G**
LIG/PDMS/1 wt.% Triton	2.1804	0.1211
LIG/PDMS/5 wt.% Triton	1.3048	0.1219
LIG/PDMS/10 wt.% Triton	0.9214	0.0947
LIG/PDMS/20 wt.% Triton	0.7811	0.5891
LIG/PDMS/30 wt.% Triton	0.6909	0.5086

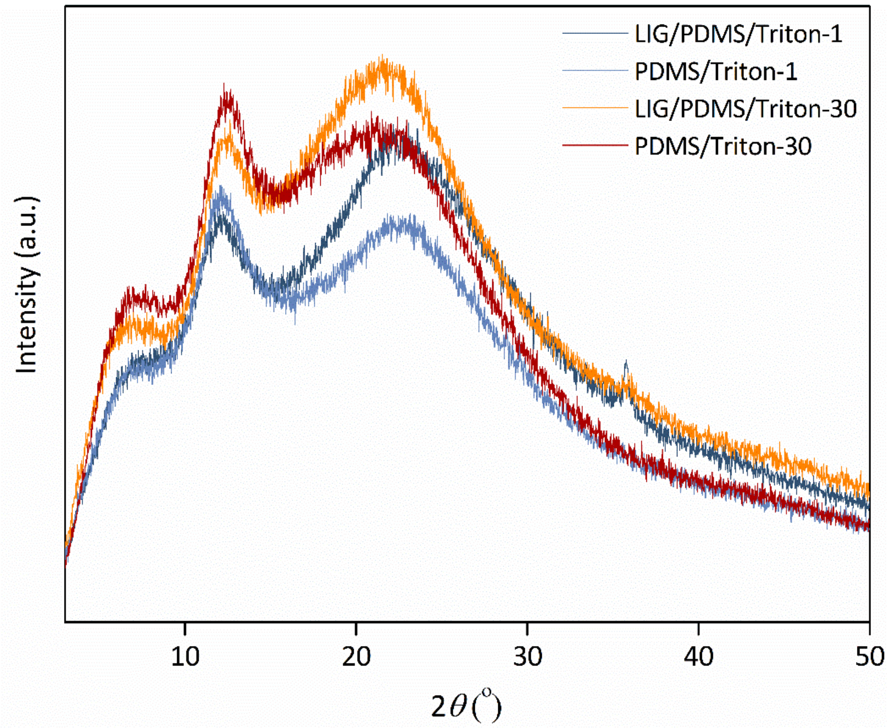

Figure S4. XRD patterns of selected PDMS/Triton and LIG/PDMS/Triton materials.

A

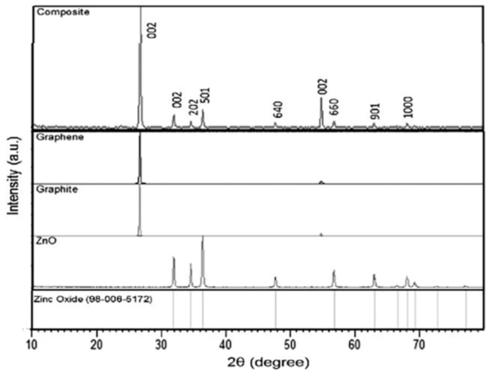

B

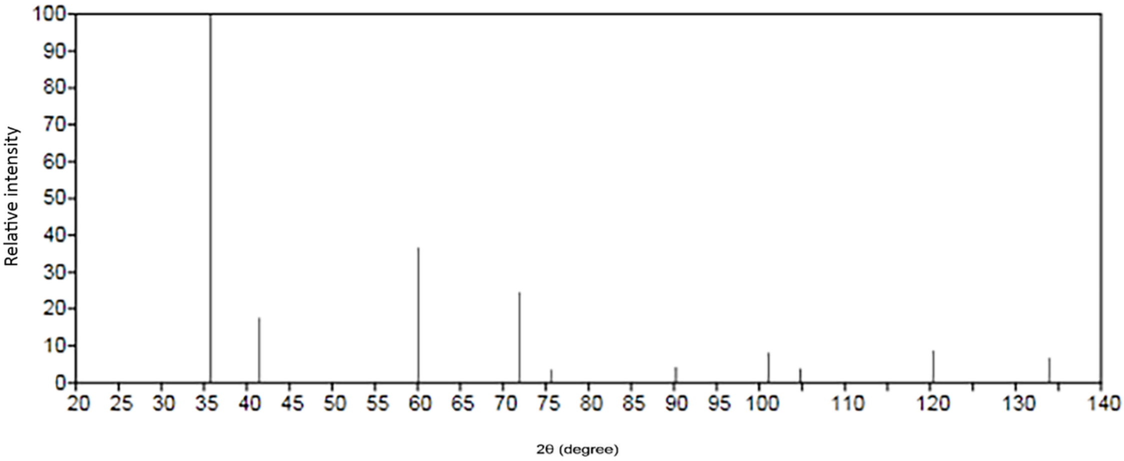

Figure S5. XRD patterns of graphene and SiC cards from instrument data.

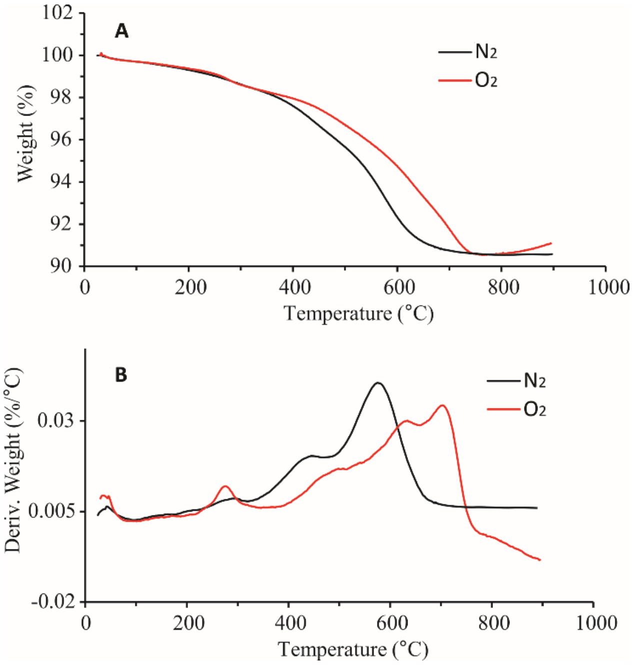

Figure S6. a) TGA and b) dTG curves of LIG in nitrogen and oxygen atmospheres.

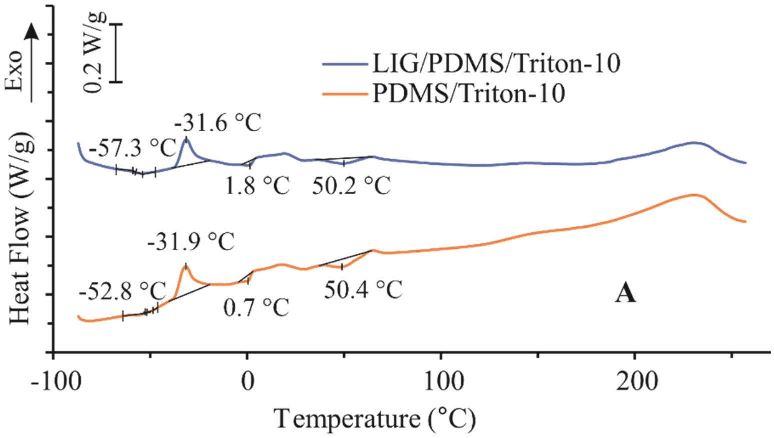

Figure S7. DSC curves of PDMS/Triton-10 and LIG/PDMS/Triton-10 materials with glass transition (T_g) and temperature maximum (T_m) of the thermal transitions (the first heating scan).

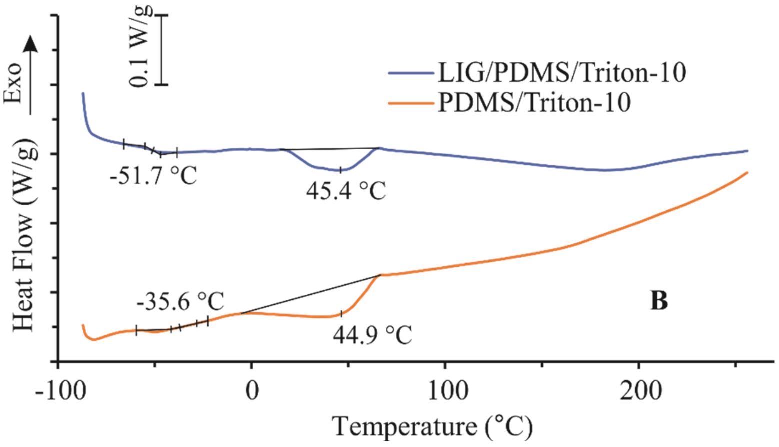

Figure S8. DSC curves of PDMS/Triton-10 and LIG/PDMS/Triton-10 materials with glass transition (T_g) and temperature maximum (T_m) of the thermal transitions (the second heating scan).

Table S4. DSC results of PDMS/Triton composite films (first heating scan)

Material	T_g (°C)	ΔH_1 (J/g)	T_{o1} (°C)	T_{m1} (°C)	T_{end1} (C)°	ΔH_2 (J/g)	To_2 (°C)	T_{m2} (C)°	T_{end2} (C)°	ΔH_3 (J/g)	T_{o3} (°C)	T_{m3} (°C)	T_{end3} (°C)
Pure PDMS	-	-	-	-	-	-	-	-	-	1.36	21.38	41.08	60.65
PDMS/5 wt.% Triton	-	-	-	-	-	-	-	-	-	1.01	38.14	50.64	62.57
PDMS/10 wt.% Triton	-52.80	5.63	-36.80	-31.91	-18.61	1.26	-2.61	0.70	2.39	6.25	19.25	50.40	62.05
PDMS/20 wt.% Triton	-58.75	11.29	-36.51	-31.84	-12.33	21.82	-2.99	0.52	2.75	1.71	38.85	50.24	62.87
PDMS/30 wt.% Triton	-58.89	16.09	-36.78	-31.88	-11.92	2.15	-6.89	0.40	3.17	0.77	47.64	52.79	62.04

Table S5. DSC results of PDMS/Triton composite films (second heating scan)

Material	T_g (°C)	ΔH_1 (J/g)	T_{o1} (°C)	T_{m1} (°C)	T_{end1} (°C°)	ΔH_2 (J/g)	T_{o2} (°C)	T_{m2} (°C)	T_{end2} (°C)	ΔH_3 (J/g)	T_{o3} (°C)	T_{m3} (°C)	T_{end3} (°C)
Pure PDMS	-	-	-	-	-	-	-	-	-	3.60	22.56	49.75	62.27
PDMS/5 wt.% Triton	-	-	-	-	-	-	-	-	-	3.53	20.63	47.97	63.48
PDMS/10 wt.% Triton	-35.64	-	-	-	-	-	-	-	-	7.31	8.12	44.94	63.10
PDMS/20 wt.% Triton	-53.86	-	-	-	-	-	-	-	-	6.79	17.38	45.50	62.89
PDMS/30 wt.% Triton	-58.33	3.72	-37.19	-31.87	-14.88	1.94	-8.09	-1.51	5.96	19.5	5.65	48.40	62.32

Table S6. DSC results of LIG/PDMS/Triton materials (first heating scan)

Material	T_g (°C)	ΔH_1 (J/g)	T_{o1} (°C)	T_{m1} (°C)	T_{end1} (°C)	ΔH_2 (J/g)	T_{o2} (°C)	T_{m2} (°C)	T_{end2} (°C)	ΔH_3 (J/g)	T_{o3} (°C)	T_{m3} (°C)	T_{end3} (°C)
Triton	-60.59	71.68	-40.40	-36.76	-13.01	14.34	-4.67	0.98	5.23	7.84	47.69	64.05	64.34
LIG/PDMS/5 wt.% Triton	-	-	-	-	-	-	-	-	-	1.01	38.14	53.82	61.17
LIG/PDMS/10 wt.% Triton	-57.54	4.85	-36.26	-31.52	-5.7	1.43	-5.71	1.80	3.78	2.62	32.77	50.02	61.62
LIG/PDMS/20 wt.% Triton	-59.06	6.81	-36.61	-32.12	-21.19	5.09	-21.19	-5.9	5.56	1.43	41.12	51.89	61.72
LIG/PDMS/30 wt.% Triton	-58.5	13.49	-37.16	-32.89	-2.23	4.60	-2.23	-1.94	7.33	0.84	45.17	52.37	62.38

Table S7. DSC results of LIG/PDMS/Triton materials (second heating scan)

**Material**	**T_g (°C)**	**ΔH_1 (J/g)**	**T_{o1} (°C)**	**T_{m1} (°C)**	**T_{end1} (°C)**	**ΔH_2 (J/g)**	**T_{o2} (°C)**	**T_{m2} (°C)**	**T_{end2} (°C)**	**ΔH_3 (J/g)**	**T_{o3} (°C)**	**T_{m3} (°C)**	**T_{end3} (°C)**
Triton	-55.91	-	-	-	-	1.02	-24.62	-1.67	-6.55	11.14	39.48	47.90	64.86
LIG/PDMS/5 wt.% Triton	-	-	-	-	-	-	-	-	-	6.46	17.44	48.13	63.52
LIG/PDMS/10 wt.% Triton	-51.70	-	-	-	-	-	-	-	-	5.34	19.41	45.43	62.48
LIG/PDMS/20 wt.% Triton	-53.67	-	-	-	-	-	-	-	-	6.08	18.56	46.99	63.05
LIG/PDMS/30 wt.% Triton	-58.01	3.91	-37.43	-31.84	-1.95	1.95	-16.15	-1.47	-5.06	5.42	20.48	48.04	62.60

Thermogravimetry (TGA) results of powdered LIG scraped from LIG/PDMS/30 wt.% Triton in the nitrogen and oxygen atmosphere

In Figure S6, the (a) TGA and (b) dTG curves of the LIG in the N_2 and O_2 atmospheres are shown. These data show that LIG underwent several weight losses in the temperature range from 25 to 800 °C. In the nitrogen atmosphere, there are evidently three weight losses, and in the oxygen atmosphere, the third mass loss became a complex and was composed of two overlapped mass losses that are visible on TGA and dTG curves. Considering the temperatures of the beginning of thermal degradation (in N_2 this was 439 °C and in O_2, this was 519 °C) and mass residues (in N_2 at 700 °C this was 91%, and in O_2 at 700 °C was 91%), it is evident that LIG is more thermostable in the oxygen atmosphere.

As, to the best of our knowledge, there was no information available in the literature on the thermal stability and decomposition of LIG obtained by thermogravimetric analysis, these measured thermal properties were compared with known thermal characteristics of graphene oxide (GO). Farivar et al. [65] explored the influence of particle size on the thermogravimetric behavior of GO. Farivar et al. [65] found that the key mass loss events of GO can be explained based on their decomposition temperatures: <100 °C for water elimination, 100–360 °C due to the removal of oxygen functional groups, and 360–1000 °C can be linked to the oxidative pyrolysis of carbon framework. Those thermal characteristics can be applied to the LIG obtained in this current work. As was found by Farivar et al. [65], the first and second DTG peaks, representing the loss of water and oxygen functional groups events, respectively, are also applicable reasons to explain the first and second DTG peaks obtained in this current work for LIG. Also, the third mass loss step of GO, being the only DTG peak found in graphene and graphite samples [65], was similar to that found in the LIG studied by us; in our current work, this peak can, therefore, be ascribed to the combustion of carbon in the applied air and oxygen conditions.
